# Neonatal acute kidney injury: Reassessing diagnostic criteria for acute kidney injury in neonates with hypoxic-ischaemic encephalopathy undergoing therapeutic hypothermia

**DOI:** 10.1007/s00467-026-07469-2

**Published:** 2026-07-22

**Authors:** Þórdís Kristinsdóttir, Milan Chromek, Mats Blennow, Katarina Robertsson Grossmann

**Affiliations:** 1 Department of Paediatrics, Karolinska University Hospital, Stockholm, Sweden; 2 Department of Clinical Science, Intervention and Technology, Division of Paediatrics, Karolinska Institutet, Nobels väg 6 171 77, Stockholm, Sweden; 3 Department of Paediatric Nephrology, Karolinska University Hospital, Stockholm, Sweden; 4 Department of Neonatology, Karolinska University Hospital, Stockholm, Sweden

**Keywords:** Acute kidney injury, Hypoxic–ischaemic encephalopathy, Short term outcome, Diagnostic criteria, Hypothermia treatment

## Abstract

**Background:**

Acute kidney injury (AKI) remains common in neonates with hypothermia-treated hypoxic–ischaemic encephalopathy (HIE). Although the modified neonatal KDIGO (nKDIGO) criteria form the diagnostic standard, their reliance on serum creatinine (SCr) increase and urine output (UO) is particularly challenging in early onset neonatal AKI. The Gupta definition, based on insufficient SCr-decline, has been proposed to identify neonates with clinically significant AKI uncaptured by nKDIGO.

**Methods:**

This retrospective cohort included 55 near-term/term neonates with hypothermia-treated HIE in Stockholm (2007–2009). Applying nKDIGO and Gupta criteria, infants were categorised as nKDIGO-AKI, Gupta-AKI, or no AKI. Primary outcome was AKI-incidence, secondary outcomes included mortality, organ support, length of stay, and signs of possible impaired kidney function at 10–12 years.

**Results:**

In our cohort, 31 (56%) suffered AKI: 17 (31%) fulfilled both definitions, 7 (13%) Gupta-only, and 7 (13%) nKDIGO-only. Need for inotropic support was more common among infants with nKDIGO-AKI compared to those without nKDIGO-AKI (*p* = 0.03). In a subanalysis, a significantly larger proportion of infants with Gupta-AKI needed inotropic support compared to those without Gupta-AKI (*p* = 0.03). Gupta-AKI was associated with a significantly higher cystatin C at 10–12 years (*p* = 0.03) compared to those without Gupta-AKI.

**Conclusions:**

The Gupta definition identified additional infants with AKI beyond nKDIGO. For both definitions, a larger proportion of infants needed inotropic support in the AKI-group compared to those without AKI. Children with a history of Gupta-AKI had a significantly higher cystatin C in early adolescence compared to those without. Incorporating Gupta criteria may improve identification of infants with significant AKI.

**Graphical Abstract:**

A higher resolution version of the Graphical abstract is available as Supplementary information

**Figure:**
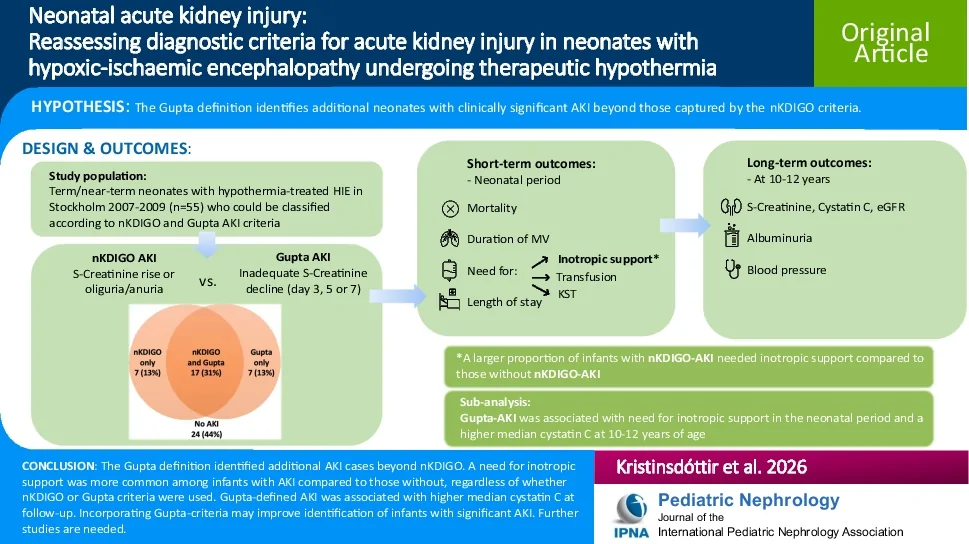

**Supplementary Information:**

The online version contains supplementary material available at https://doi.org/10.1007/s00467-026-07469-2.

## Introduction

Acute kidney injury (AKI) remains a frequent complication in neonates with hypoxic–ischaemic encephalopathy (HIE), with reported incidence rates of 30–40%, also in the age of therapeutic hypothermia (TH) [1–3]. Neonatal AKI is independently associated with increased mortality and prolonged hospitalisation, yet it remains under-recognised, partly due to limitations inherent to current diagnostic criteria [4, 5]. The modified neonatal KDIGO (nKDIGO) definition is currently the most widely accepted standard for diagnosing neonatal AKI. However, it relies on changes in serum creatinine (SCr)—defined as either a relative increase from baseline or an absolute rise—and on urine output (UO), both of which pose interpretive challenges in neonates. In near-term and term infants, SCr at birth reflects maternal creatinine and normally declines during the first postnatal week, making it difficult to distinguish physiological adaptation from pathological kidney injury. Furthermore, in cases of perinatal asphyxia, kidney injury may occur peripartum, resulting in elevated SCr already at birth or shortly thereafter [6–8]. This can render the detection of early AKI using the SCr-criteria of the nKDIGO definition particularly difficult. In addition, UO criteria are only applicable from day 2 of life, quantification of UO may be unreliable, and neonatal AKI is frequently non-oliguric [9]. Despite these diagnostic challenges, timely recognition of neonatal AKI is of great importance. No disease-specific therapy exists once AKI is established; thus, management is largely supportive and focuses on hemodynamic optimisation and avoidance of additional kidney injury. Importantly, beyond its association with short-term adverse outcomes, AKI has been increasingly linked to subsequent chronic kidney disease (CKD) [10–12]. Follow-up studies of neonatal AKI survivors report substantial rates of later CKD, also in early childhood, although estimates vary due to heterogeneity in study design and outcome definitions [13–15]. These considerations highlight the need for improved diagnostic strategies, risk-stratification and subsequent long-term surveillance of infants affected by neonatal AKI.

Emerging evidence suggests that a failure of SCr to decline at the expected rate during the first week of life may serve as a sensitive marker of impaired kidney function in neonates [16, 17]. The Gupta definition, based on the rate of SCr decline, has identified a subset of neonates with clinically significant AKI not captured by the nKDIGO criteria but who still experience increased risks of adverse outcomes, including longer hospital stay, prolonged respiratory support, and an increased risk of the composite outcome mortality and/or moderate or severe brain MRI injury [17, 18].

Given the vulnerability of neonates with HIE and the association between AKI and both short- and long-term adverse outcomes, early and accurate identification of AKI is essential to guide clinical management and structured follow-up [19, 20]. Data specifically addressing long-term kidney function after AKI in HIE-survivors remain limited. In our population-based cohort of children with a history of hypothermia-treated HIE, we have previously shown that kidney sequelae were rare at 10–12 years of age, regardless of neonatal AKI status as per nKDIGO definition. Furthermore, our in-depth assessment of kidney health suggested that the Schwartz–Lyon equation appears to underestimate glomerular filtration rate (GFR) in this patient population [21]. Recent studies suggesting that the Gupta criteria may identify additional neonates with clinically relevant AKI and adverse short-term outcomes prompted us to re-evaluate our cohort using Gupta criteria alongside nKDIGO criteria [17, 18].

In this study, we hypothesise that also using the Gupta definition will identify additional neonates with AKI not captured by nKDIGO, potentially revealing a high-risk subgroup otherwise overlooked in clinical practice.

## Materials and methods

### Study design and population

The cohort has been described in detail previously, as have national guidelines for therapeutic hypothermia in Stockholm during 2007–2009 [3]. In the present study, the same cohort was re-evaluated for neonatal AKI also according to the Gupta criteria. Infants with sufficient SCr measurements to assess postnatal SCr-decline constituted the subcohort included in this analysis.

The study comprised assessment of short-term neonatal outcomes and long-term kidney follow-up at 10–12 years of age.

### Definitions of neonatal AKI

Infants were classified according to kidney function during the first 7 days of life:

- nKDIGO-AKI: Fulfilled nKDIGO SCr and/or UO criteria (Table 1)
- Gupta-AKI: Fulfilled Gupta criteria with insufficient SCr decline (Table 1)
- nKDIGO- and/or Gupta-AKI: Fulfilled either nKDIGO orGupta criteria, or both
- No AKI: Fulfilled neither nKDIGO nor Gupta criteria.

**Table 1:** Modified KDIGO criteria (nKDIGO) and Gupta criteria for neonatal acute kidney injury (AKI)

**nKDIGO criteria for neonatal AKI**		
**Stage**	**Serum Creatinine (SCr)**	**Urine Output (UO)**
Stage 1	Increase in SCr ≥ 26.5 µmol/L within 48 h, or 1.5–1.9 × baseline*	≤1.0 mL/kg/h for ≥24 h
Stage 2	Increase in SCr 2.0–2.9 × baseline*	≤0.5 mL/kg/h for ≥24 h
Stage 3	Increase in SCr ≥ 3.0 × baseline*, or SCr ≥ 221 µmol/L, or initiation of dialysis	≤0.3 mL/kg/h for ≥24 h
**Gupta criteria for neonatal AKI**		
Serum creatinine decline from birth:		
≤33% by day 3		
or		
≤40% by day 5		
or		
≤46% by day 7		

Note: Adapted from Ahn et al. (2024) [18]

*Within 7 days of trough

Subgrouping was based on the number of fulfilled criteria. Classification required at least two SCr values (one on day 0–1 and another on days 3, 5, or 7). UO-defined AKI required at least one day of reliably quantifiable UO. Comparisons were made first between infants with and without AKI according to the nKDIGO definition, secondly between infants with and without AKI according to the nKDIGO and/or Gupta definition. In a subanalysis, we also compared outcomes between infants meeting Gupta criteria for AKI and those who did not. Additionally, a subanalysis was conducted focusing on infants fulfilling the nKDIGO UO criteria.

### Long-term kidney follow-up (10–12 years)

The long-term follow-up of this cohort has been described previously [21]. In brief, surviving children residing in Sweden were invited to a standardised evaluation at 10–12 years of age following informed consent.

For the present study, kidney function was assessed by measuring serum creatinine using the Jaffé method, with estimated glomerular filtration rate (eGFR) calculated via the Schwartz–Lyon formula [22]. Cystatin C was measured by turbidimetry, and eGFR was calculated using the CAPA formula [23]. Children with reduced eGFR underwent confirmatory measurement of GFR by plasma iohexol clearance. First-morning urine albumin-to-creatinine ratio was analysed, with repeated testing to confirm persistent albuminuria when elevated. Anthropometrics were assessed using national growth references. Office blood pressure was measured in triplicate after rest according to national paediatric hypertension guidelines [24] and children with elevated values underwent 24-h ambulatory monitoring.

### Outcomes

#### Short-term outcomes

The primary neonatal outcome was AKI according to nKDIGO and/or Gupta definitions. Secondary short-term outcomes included neonatal mortality, duration of mechanical ventilation (MV), need for inotropic support, need for transfusion of blood products, need for kidney support therapy (KST), and length of hospital stay (LOS).

#### Long-term outcomes

Long-term outcome was evidence of impaired kidney function at 10–12 years, defined as reduced eGFR calculated using the Schwartz–Lyon or reduced cystatin C eGFR calculated by the CAPA formula and confirmed with plasma iohexol clearance, category A2 albuminuria or greater according to the KDIGO definition [25], and/or elevated blood pressure equal to or above the 90th percentile for sex, age and height.

### Statistical analysis

All analyses were conducted using JASP software (Version 0.95.4; JASP Team, 2025). Continuous variables were compared between two groups using the Mann–Whitney *U* test. Continuous variables that were not normally distributed were analysed using the Kruskal–Wallis test when comparing more than two groups. When the omnibus test was statistically significant, post hoc pairwise comparisons were performed using Dunn’s test with Bonferroni correction to adjust for multiple comparisons. Categorical variables were analysed using the chi-square test when all expected cell counts exceeded five. For smaller groups with expected cell counts below five, Fisher’s exact test was applied. When comparing categorical variables across more than two groups, the chi-square test was initially used; however, due to small subgroup sizes in subanalysis, Fisher’s exact test was employed for pairwise comparisons to enhance accuracy. Statistical significance was defined as *p* < 0.05.

### Ethics

The study was approved by the Ethics Review Board in Sweden (2019–01447).

## Results

### Patient characteristics

Of the original 65 patients, 55 patients had sufficient SCr measurements obtained for AKI-classification according to both nKDIGO and Gupta criteria and were included in the final analysis. Ten patients (15.4%) were excluded due to insufficient serum creatinine (SCr) measurements for Gupta classification. Of the 55 included patients, five lacked an admission SCr but had a valid day 1 measurement used as baseline for analysis. Patient characteristics and demographics for the final cohort are shown in Table 2. The nKDIGO-AKI group had significantly lower Apgar scores at both 5 and 10 min compared to the no nKDIGO-AKI group (*p* = 0.02 and *p* = 0.03, respectively). No statistically significant difference was found between these groups for sex, gestational age (GA), birthweight, pH value, base excess, age at start of cooling or severity of HIE. When also incorporating patients who met the Gupta criteria for AKI, the nKDIGO- and/or Gupta-AKI group had significantly lower Apgar score at 10 min (*p* = 0.04) and lower BE (*p* = 0.02) compared to the no AKI group. No statistically significant differences were found between these groups for sex, GA, birthweight, pH value, age at start of cooling or severity of HIE.

**Table 2:** Patient characteristics in infants with (a) nKDIGO-AKI compared to no nKDIGO-AKI and (b) nKDIGO- and/or Gupta-AKI compared to no AKI

a)			
**Characteristic**	**nKDIGO-AKI (*n* = 24)**	**No nKDIGO-AKI (*n* = 31)**	***p*-value**
Sex (boys/girls)	11/13	16/15	NS*
Gestational age (days)	282.5 (272.8–289.3; 246–295)	283.0 (274.5–288.0; 238–295)	NS**
Birth weight (g)	3625 (3289.8–3991.3; 2380–4565)	3445 (3118.0–3992.0; 2020–5828)	NS**
Apgar at 5 min	2.0 (1.0–3.3; 0–9)	4.0 (2.0–4.0; 0–10)	*p* < 0.05**
Apgar at 10 min	3.0 (2.0–5.0; 0–9)	5.5 (4.0–6.8; 1–10)	*p* < 0.05**
pH	6.9 (6.8–6.9; 6.5–7.2)	6.9 (6.8–6.9; 6.7–7.3)	NS**
Base excess (mmol/L)	−22.0 (−25.0 to −18.0; −29.0 to −10.7)	−17.4 (−20.0 to −14.3; −36.0 to −7.1)	NS**
Age at start of active cooling (hrs)	3.0 (2.3–5.3; 1.0–13.0)	4.0 (2.3–5.2; 1.0–10.0)	NS**
Degree of HIE:			NS**
Mild	2 (8.3%)	1 (3.2%)	
Moderate	17 (70.8%)	29 (93.5%)	
Severe	5 (20.8%)	1 (3.2%)	
b)			
**Characteristic**	**nKDIGO- and/or Gupta-AKI (*n* = 31)**	**No AKI (*n* = 24)**	***p*-value**
Sex (boys/girls)	14/17	13/11	NS*
Gestational age (days)	281.0 (270.5–289.0; 238–295)	284.0 (280.8–288.0; 267–295)	NS**
Birth weight (g)	3498 (3214.0–3955.0; 2376–4565)	3484 (3195.3–4325.5; 2020–5828)	NS**
Apgar at 5 min	3.0 (1.0–4.0; 0–9)	4.0 (2.0–4.0; 0–10)	NS**
Apgar at 10 min	4.0 (2.0–5.0; 0–9)	5.0 (4.0–6.0; 1–10)	*p* < 0.05**
pH	6.9 (6.7–6.9; 6.5–7.2)	6.9 (6.8–7.0; 6.7–7.3)	NS**
Base excess (mmol/L)	−21.5 (−25.0 to −17.3; −36.0 to −10.7)	−16.9 (−20.0 to −14.5; −24.0 to −7.1)	*p* < 0.05**
Age at start of active cooling (hrs)	3.8 (2.4–5.5; 1.0–13.0)	3.8 (2.1–4.8; 1.0–10.0)	NS**
Degree of HIE:			NS**
Mild	3 (9.7%)	0 (0%)	
Moderate	23 (74.2%)	23 (95.8%)	
Severe	5 (16.1%)	1 (4.2%)	

Continuous variables are presented as median (interquartile range [Q1–Q3]; minimum–maximum)

Categorical variables are shown as n (%)

*Chi-square test. **Mann–Whitney U test

### Excluded patients

Among the 10 patients (15.4%) excluded from further analyses due to insufficient SCr measurements for classification according to Gupta criteria, five met nKDIGO-AKI criteria. Of these, one met the nKDIGO SCr criteria, two met the UO criteria, and two fulfilled both SCr and UO criteria. Among them, three infants had stage 1 AKI, one had stage 2, and one had stage 3. The remaining five patients did not meet any nKDIGO-AKI criteria. When comparing patient characteristics and demographics for the excluded patients to the included patients, the excluded patients had significantly worse HIE score at the first day of life (*p* = 0.03), where four of the five infants in the nKDIGO group had severe HIE. No significant difference was found between the included and excluded patients for other patient characteristics or demographics. Ultimately, only four of the excluded patients participated in the long-term follow up, where only one had history of neonatal AKI, and none of them had evidence of impaired kidney function at the age of 10–12 years old.

### AKI incidence

**Fig. 1:**
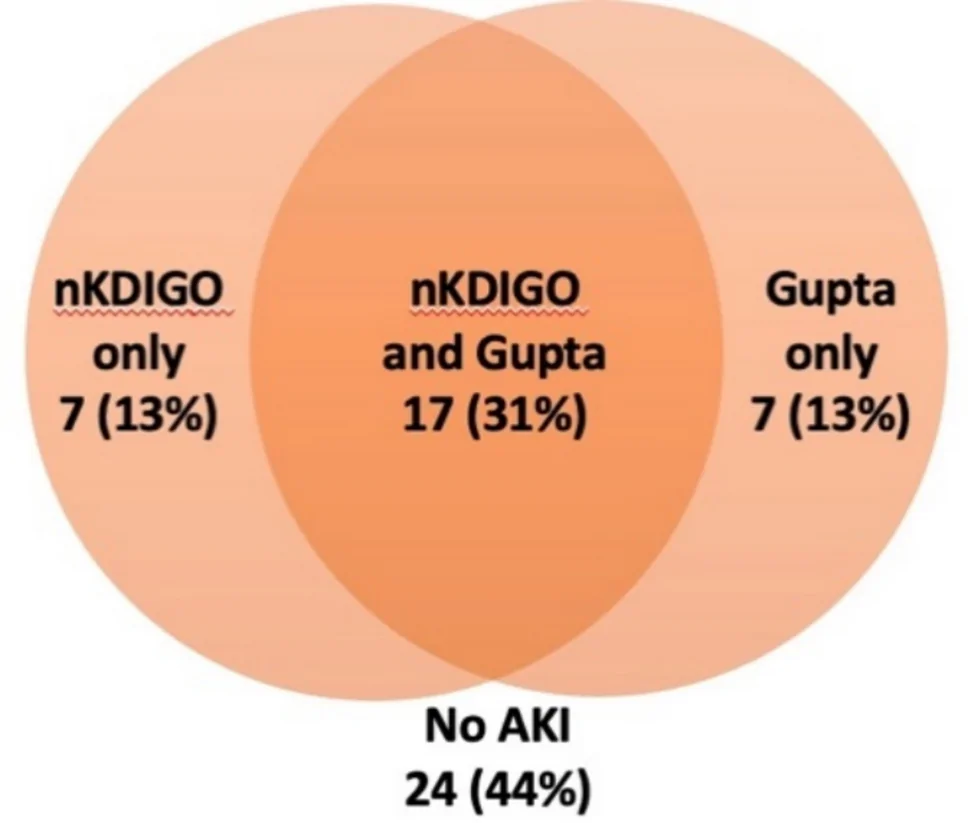
Venn diagram of AKI classification

Among the 55 included infants, 31 (56.4%) met criteria for AKI. Of these, 24 (43.6%) met nKDIGO criteria, 24 (43.6%) met Gupta criteria, 17 (30.9%) met both Gupta and nKDIGO criteria, whereas 24 (43.6%) patients met neither. One patient with declining SCr after initiation of kidney support therapy (KST) on day 3 of life was classified as Gupta positive since a need for KST is considered AKI regardless of definition. Applying the Gupta criteria identified seven additional patients with AKI (12.7%) not captured by any nKDIGO criteria (Gupta-only AKI). Figure 1 presents a Venn diagram and Table 3a contingency table of the different AKI groups.

**Table 3:** Contingency table of AKI classification

	Gupta (+)	Gupta (–)	Total
KDIGO (+)	17 (30.9%)	7 (12.7%)	24 (43.6%)
KDIGO (–)	7 (12.7%)	24 (43.6%)	31 (56.4%)
Total	24 (43.6%)	31 (56.4%)	55 (100%)

Further division into AKI subgroups showed that of the 24 (43.6%) infants who met the nKDIGO criteria, 7 (12.7%) met both SCr and UO criteria, 6 (10.9%) met only SCr, and 11 (20.0%) met only UO criteria, respectively. Seven patients met neither Gupta nor nKDIGO SCr criteria but only the nKDIGO UO criteria. Gupta criteria were met by 24 infants (43.6%), including 7 (12.7%) who did not meet nKDIGO. Among the 17 infants who met both definitions, 4 (7.3%) fulfilled the Gupta criteria and the nKDIGO UO criteria but did not meet the nKDIGO SCr criteria.

### Short-term outcomes

Two deaths (8.3%) occurred in the nKDIGO-AKI group, and one (3.2%) in the no nKDIGO-AKI group. This difference was not statistically significant (*p* = 0.41). A significantly larger proportion of infants in the nKDIGO-AKI group needed inotropic support compared to those without nKDIGO-AKI (*p* = 0.03). For all other binary secondary short-term outcomes, no significant differences were found between these groups. For the continuous variables of duration of MV and LOS, there was no significant difference (*p* = 0.16 and *p* = 0.27, respectively). Despite higher means in the nKDIGO-AKI group, no pairwise post hoc differences were significant. Results are summarised in Table 4a.

Two deaths (6.5%) occurred in the nKDIGO- and/or Gupta-AKI group, and one (4.2%) in the no-AKI group. This difference was not statistically significant (*p* = 0.71). No deaths occurred among infants with Gupta-only AKI. Including infants with AKI according to Gupta criteria in the AKI group resulted in no statistically significant differences in any secondary short-term outcomes when comparing infants with nKDIGO- and/or Gupta-AKI to those without AKI. For the continuous variables of duration of MV and LOS, there was no significant difference (*p* = 0.57 and *p* = 0.89, respectively). Results are summarised in Table 4b.

**Table 4:** Summary of descriptive statistics for short-term secondary outcomes in (a) infants with nKDIGO-AKI compared to no nKDIGO-AKI and (b) nKDIGO- and/or Gupta-AKI compared to no AKI

a)			
**Outcome variable**	**nKDIGO-AKI (*n* = 24)**	**No nKDIGO-AKI (*n* = 31)**	***p*-value**
LOS (days)	13.0 (9.8–15.3; 3.0–37.0)	10.0 (8.0–16.0; 6.0–57.0)	NS*
Duration of MV (hours)	22.0 (0.3–105.0; 0.0–427.3)	1.7 (0.0–36.0; 0.0–192.0)	NS*
Need for transfusion of blood products	22 (91.7%)	23 (76.7%)	NS**
Need for KST	1 (4.2%)	0 (0%)	NS**
Need for inotropic support	14 (63.6%)	9 (32.1%)	*p* < 0.05**
Neonatal mortality	2 (8.3%)	1 (3.2%)	NS**
b)			
**Outcome variable**	**nKDIGO- and/or Gupta-AKI (*n* = 31)**	**No AKI (*n* = 24)**	***p*-value**
LOS (days)	11.0 (9.0–15.0; 6.0–57.0)	10.0 (8.0–18.0; 6.0–57.0)	NS*
Duration of MV (hours)	12.0 (0.2–96.0; 0.0–473.0)	2.0 (0.2–42.0; 0.0–192.0)	NS*
Need for transfusion of blood products	27 (87.1%)	18 (78.3%)	NS**
Need for KST	1 (3.2%)	0 (0%)	NS**
Need for inotropic support	16 (57.1%)	7 (31.8%)	NS**
Neonatal mortality	2.0 (6.5%)	1 (4.2%)	NS**

Continuous variables are presented as median (interquartile range [Q1–Q3]; minimum–maximum)

Categorical variables are shown as n (%)

*Chi-square test. **Mann–Whitney U test

In a subanalysis, we compared short-term outcomes between infants with Gupta-AKI (*n* = 24)and those with no Gupta-AKI (*n* = 31). In this analysis, the proportion of infants needing inotropic support was significantly higher among infants with Gupta-AKI compared to those without (*p* = 0.03). Notably, all infants with Gupta-AKI who simultaneously met only the UO criteria of the nKDIGO definition (*n* = 4) required inotropic support, a frequency that differed significantly from other infants (*p* = 0.02) and was even more pronounced when compared to those without AKI (*p* = 0.01). No significant differences were found for other short-term outcomes, including mortality, duration of MV, need for transfusion of blood products, KST, or LOS.

To further assess the clinical relevance of the Gupta versus nKDIGO criteria for neonatal AKI, we also conducted a subgroup analysis dividing the cohort into four groups: No AKI (*n* = 24), nKDIGO- and Gupta-AKI (*n* = 17), Gupta-only AKI (*n* = 7), and nKDIGO-only AKI (*n* = 7). A statistically significant larger proportion of infants in the combined nKDIGO- and Gupta-AKI group needed inotropic support compared to the no AKI group (*p* = 0.02). No significant differences were found across groups in terms of any other short-term outcome when comparing these four groups. When further evaluating the infants who met the nKDIGO UO criteria, regardless of simultaneously meeting Gupta- and/or nKDIGO SCr criteria (*n* = 18), there was no significant difference in any short-term outcome when compared to others. Similarly, infants who fulfilled only the nKDIGO UO criteria (*n* = 7), without meeting any SCr criteria, also showed no significant differences in short-term outcomes when compared to others.

### Long-term outcomes

Of the original 55 children in the cohort, 42 (76%) participated in the follow-up assessment at 10–12 years of age. Four children had died (three prior to discharge from the neonatal unit and one in early childhood), four were lost to follow-up due to relocation abroad, four families declined participation, and one child was excluded because of a genetic syndrome. Blood samples were obtained from 37 children, 39 provided a morning urine sample, and blood pressure measurement was successful in all but one child due to severe cerebral palsy with involuntary movements.

**Fig. 2:**
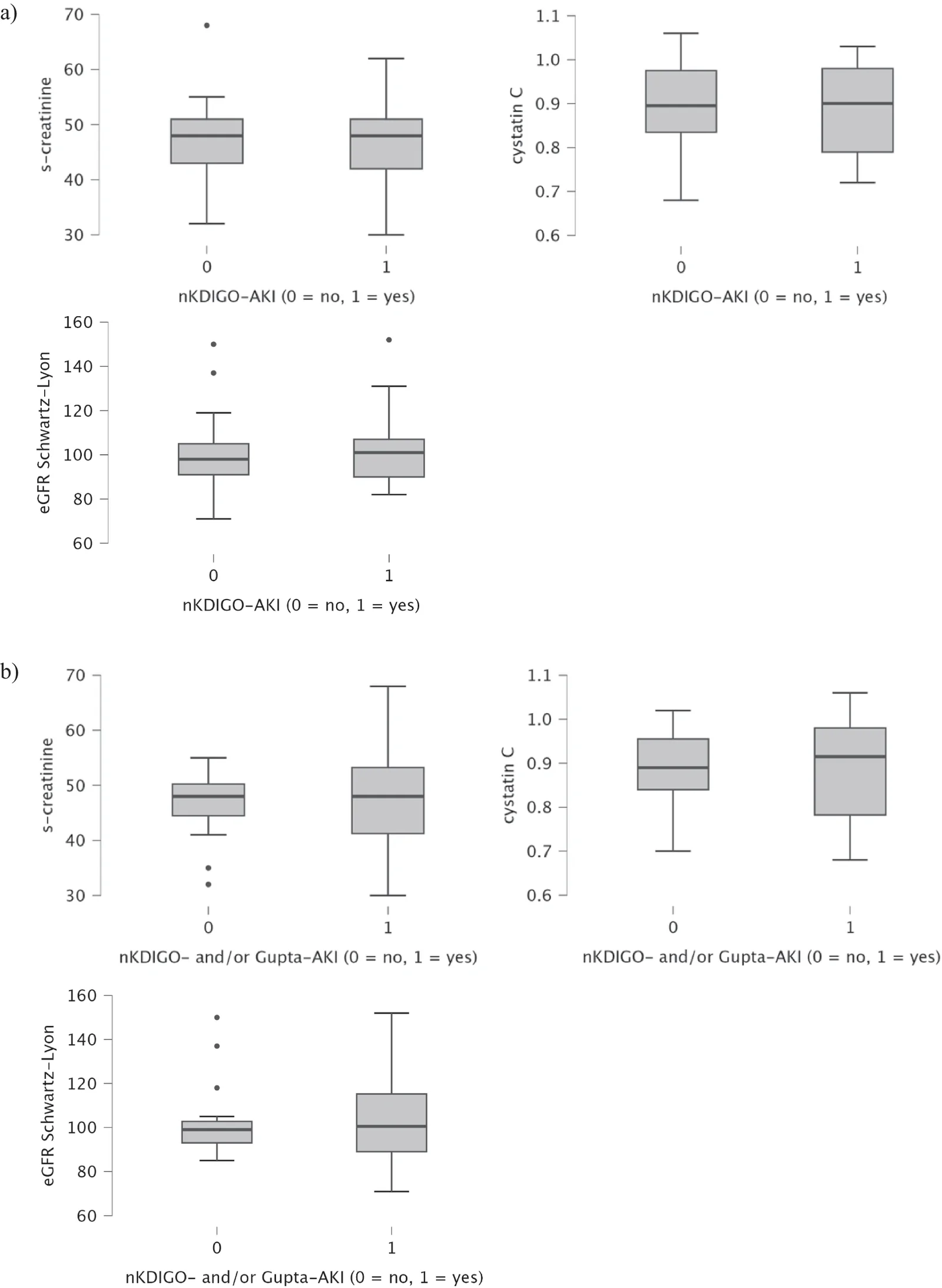
Boxplots illustrating serum creatinine, cystatin C, and Schwartz-Lyon estimated eGFR at 10–12-year follow-up in children with a history of **a** nKDIGO-AKI compared to no nKDIGO-AKI and **b** nKDIGO- and/or Gupta-AKI compared to no AKI. Note: Boxplots display continuous variables as median, interquartile range (Q1–Q3), and minimum–maximum values

Median SCr, cystatin C, and estimated GFR from SCr using the Schwartz–Lyon formula were all within normal range and did not differ significantly between children with a history of neonatal nKDIGO-AKI to those without nKDIGO-AKI (*p* = 0.85, 0.95, and 0.58 for SCr, cystatin C, and eGFR, respectively), nor when comparing children with nKDIGO- and/or Gupta-AKI to those without AKI (*p* = 0.64, 0.80, and 0.89, respectively). Figure 2 displays boxplots illustrating SCr, cystatin C, and Schwartz–Lyon eGFR values in the different groups (shown as the median, interquartile range (IQR), and minimum to maximum values). Only one child had reduced kidney function confirmed by iohexol clearance. This child initially had a reduced cystatin C-based eGFR (81) that normalised on repeated testing; however, given a history of neonatal KST and thus AKI according to both nKDIGO and Gupta criteria, measured GFR was performed and was slightly reduced (88). Another child had persistently reduced cystatin C-based eGFR on repeated testing (85 and 80) but iohexol clearance was normal and did not confirm impaired kidney function. This child had met Gupta criteria but not nKDIGO AKI criteria. The incidence of confirmed reduction in kidney function GFR < 90 ml/min/1.73 m^2^ did not differ significantly between groups.

Four additional children—two with a history of nKDIGO-AKI and two without nKDIGO-AKI—had a slight reduction in cystatin C-based eGFR on initial testing. In three of these children, eGFR normalised upon repeated assessment; the fourth child did not undergo repeated testing, and confirmation was therefore not possible.

One out of 39 children (2.6%) had KDIGO category A2 albuminuria. This child fulfilled both nKDIGO and Gupta AKI criteria. This did not yield a statistically significant difference between any of the neonatal AKI comparison groups.

Three of 41 children (7.3%) had elevated office blood pressure at follow-up; two had normal 24-h ambulatory blood pressure monitoring and one had high normal blood pressure. One of these children had fulfilled both nKDIGO and Gupta AKI criteria, while two had no history of neonatal AKI. There was no statistically significant difference between comparison groups. None met criteria for hypertension. Findings are summarised in Table 5.

**Table 5:** Summary of descriptive statistics for long-term outcomes in children with a history of (a) nKDIGO-AKI compared to no nKDIGO-AKI, and (b) nKDIGO- and/or Gupta-AKI compared to no AKI

a)			
**Outcome variable**	**nKDIGO-AKI (*n* = 24)**	**No nKDIGO-AKI (*n* = 31)**	***p*-value**
Cystatin C (mg/L)	0.9 (0.8–1.0; 0.7–1.0)	0.9 (0.8–1.0; 0.7–1.1)	NS*
Serum creatinine (µmol/L)	48.0 (42.0–51.0; 30.0–62.0)	48.0 (43.0–51.0; 32.0–68.0)	NS*
Schwartz–Lyon estimated GFR (mL/min/1.73 m^2^)	101.0 (90.0–107.0; 82.0–152.0)	98.0 (91.0–105.0; 71.0–150.0)	NS*
GFR < 90 ml/min/1.73 m^2^ confirmed by iohexol clearance	1 (5.9%)	0 (0%)	NS**
Proteinuria (UACR ≥ 3 mg/mmol)	1 (6.7%)	0 (0%)	NS**
Elevated office BP	1 (5.6%)	2 (9.1%)	NS**
b)			
**Outcome variable**	**nKDIGO- and/or Gupta-AKI (*n* = 31)**	**No AKI (*n* = 24)**	***p*-value**
Cystatin C (mg/L)	0.9 (0.8–1.0; 0.7–1.1)	0.9 (0.8–1.0; 0.7–1.0)	NS*
Serum creatinine (µmol/L)	48.0 (41.3–53.5; 30.0–68.0)	48.0 (44.5–50.3; 32.0–55.0)	NS*
Schwartz–Lyon estimated GFR (mL/min/1.73 m^2^)	100.5 (89.0–115.3; 71.0–152.0)	99.0 (93.0–102.8; 85.0–150.0)	NS*
GFR < 90 ml/min/1.73 m^2^ confirmed by iohexol clearance	1 (4.5%)	0 (0%)	NS**
Proteinuria (UACR ≥ 3 mg/mmol)	1 (2.6%)	0 (0%)	NS**
Elevated office BP	1 (4.2%)	2 (12.5%)	NS**

Note. Continuous variables are presented as median (interquartile range [Q1–Q3]; minimum–maximum)

Categorical variables are shown as n (%)

*Chi-square test. **Mann–Whitney U test

**Fig. 3:**
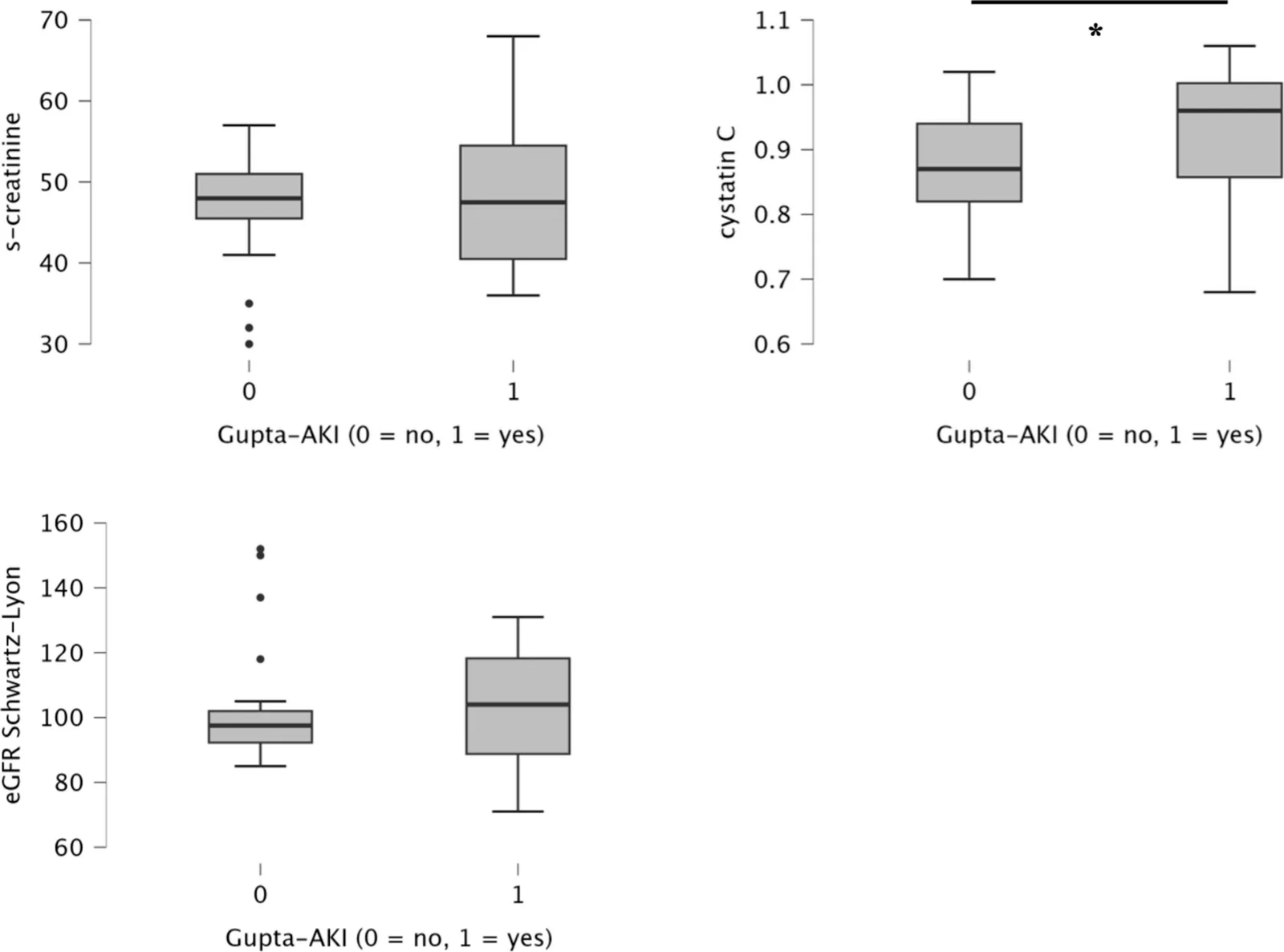
Boxplots illustrating serum creatinine, cystatin C, and Schwartz–Lyon estimated eGFR (Schwartz–Lyon) at 10–12-year follow-up for children with history of Gupta-AKI compared to those without. Note: Boxplots display continuous variables as median, interquartile range (Q1–Q3), and minimum–maximum values

As was done for the short-term outcomes, a subanalysis was performed for long-term outcomes between children with and without a history of neonatal Gupta-AKI. In this analysis, median SCr and eGFR using the Schwartz–Lyon formula were similar between groups. However, children with a history of neonatal Gupta-AKI exhibited significantly higher cystatin C compared to those without Gupta-AKI (*p* = 0.03). The incidence of confirmed GFR < 90 ml/min/1.73 m^2^, albuminuria, or elevated blood pressure did not differ significantly between groups. Boxplots in Fig. 3 illustrate median SCr, cystatin C, and Schwartz–Lyon estimated eGFR values in children with and without a history of neonatal Gupta-AKI (shown as the median, IQR, and minimum to maximum values).

## Discussion

In this retrospective cohort of 55 neonates with HIE treated with therapeutic hypothermia, we investigated the incidence of neonatal AKI diagnosis when applying the Gupta criteria—based on insufficient decline in serum creatinine—alongside the standard modified nKDIGO criteria. Doing so identified seven additional AKI cases, bringing the total incidence to 56.4%. In contrast to our previous report, we observed that a need for inotropic support was more common among infants with nKDIGO-AKI compared to those without nKDIGO-AKI. However, the previously reported increased mortality and higher proportion of infants requiring blood transfusions when comparing nKDIGO-AKI to no nKDIGO-AKI were not observed in this subcohort [3]. No other significant differences in short- or long-term outcomes were found between these groups, whether AKI was defined by nKDIGO criteria alone or in combination with Gupta criteria. Similarly, a larger proportion of infants with Gupta-AKI required inotropic support compared to those without Gupta-AKI, with no differences observed in other short-term outcomes. Upon assessment of kidney outcomes at age 10–12, evidence of long-term kidney sequelae was rare. Although still within normal range, the group with a history of Gupta-AKI had significantly higher median cystatin C-levels compared to those without.

Our findings align with previous studies that have found that Gupta-based definitions may detect neonates with impaired kidney function otherwise missed by the nKDIGO criteria [17, 18]. However, unlike the report by Ahn et al., the small number of infants with Gupta-only AKI in our cohort precluded meaningful analysis of this specific subgroup. Nonetheless, we found that infants with Gupta-AKI—regardless of whether simultaneously meeting nKDIGO criteria—had a significantly higher proportion requiring inotropic support compared to those without Gupta-AKI. Similarly, Ahn et al., reported that a significantly higher proportion of infants with Gupta-only AKI required inotropic support on day 1 compared with infants without AKI; however, this was described as a baseline characteristic rather than an outcome [18].

In the 10–12-year follow-up of our cohort, signs of chronic kidney dysfunction were uncommon and did not differ significantly between any of the neonatal AKI comparison groups. Only isolated cases of borderline reduction in eGFR, albuminuria or elevated blood pressure were observed. In a subanalysis, children with a history of Gupta-AKI had significantly higher cystatin C levels at follow-up compared to those without a history of Gupta-AKI; despite a large overlap between individuals who had met both nKDIGO- and Gupta-AKI criteria, this difference was not observed in the primary comparison. These findings suggest that the Gupta criteria possibly identify a more subtle acute impairment of kidney function in neonates that may still have potential long-term clinical significance. However, considering that eGFR and cystatin C remained within the normal range, these results should only be considered hypothesis-generating and require confirmation through larger cohorts, ideally with follow-up extending into later adolescence or even adulthood.

The scarcity of long-term kidney sequelae in our cohort contrasts with several longitudinal studies reporting higher rates of CKD markers after neonatal AKI. To date, there are very few reports on long-term kidney outcomes following neonatal AKI in the setting of hypothermia-treated HIE. Most studies on long-term kidney outcomes have focused on other neonatal patient populations [13]. In a follow-up study of 95 children 2–12 years after neonatal AKI (according to the nKDIGO criteria), one third of children demonstrated subclinical kidney dysfunction—such as proteinuria (13%), office hypertension (13%) or hyperfiltration (10%)—and 48% had abnormal ambulatory blood pressure monitoring. Of note, many of these patients had a history of premature birth, low birth weight and critical illness in the neonatal period [15]. In another study of 34 children with a history of very low birth weight who were evaluated at 3–7 years of age, those with a history of neonatal AKI had a significantly higher risk of kidney dysfunction compared with those without AKI (65% vs. 14%, RR 4.5), with 26% of the total cohort demonstrating reduced eGFR based on cystatin C [14]. A prospective longitudinal cohort study of 58 full-term neonates who underwent congenital heart disease surgery found that 58% experienced surgery-associated AKI. At 6 years, the prevalence of CKD and hypertension was 17% and 30%, respectively, with an additional 15% exhibiting elevated blood pressure. Notably, surgery-associated AKI was not associated with CKD or hypertension in this cohort [26]. Regarding the scarcity of CKD in our cohort, we can only hypothesise whether that can be in part explained by a protective effect of therapeutic hypothermia or the absence of prematurity-related low nephron endowment and major comorbidities that characterise the higher-risk neonatal populations mostly studied previously. We can further speculate that even assessment in early adolescence may be too early to detect signs of CKD, and that follow-up should continue also into adulthood. Neonatal AKI may also impact other organ systems, both short- and long-term [19, 27]. Cavallin et al. found worse neurological outcomes at 24 months in infants with HIE and nKDIGO-defined AKI, however, kidney function was not assessed at the time of follow-up [20]. Recently, a panel of experts recommended routine inclusion of kidney outcomes in neonatal trials, citing risks of early CKD and multi-organ morbidity following AKI [28]. In 2024, a multidisciplinary workgroup published a consensus guideline for neonates with history of AKI according to nKDIGO that includes a discharge kidney health evaluation, as well as subsequent follow up at a certain age [29]. These recommendations have yet to be implemented into regular clinical practice in Sweden.

Our study has several limitations. It was a single-centre retrospective analysis with a modest cohort size, reducing statistical power and resulting inference, particularly for long-term outcomes due to the low event rate. Although data completeness was generally high, some missing data may have affected the results. Notably, 10 infants were excluded for the purpose of this study due to insufficient SCr measurement for Gupta classification—five of whom met the nKDIGO-AKI definition. Importantly, four of these five infants had grade 3 HIE and died during the neonatal period. Their exclusion may have introduced bias, particularly affecting mortality outcomes and could explain why we did not observe the previously reported significant difference in mortality between infants with and without nKDIGO-AKI as in the original cohort [3]. This might also account for the lack of mortality difference in the comparison between nKDIGO- and/or Gupta-AKI versus no AKI groups. Furthermore, the exclusion of these more severely ill patients may have impacted the assessment of other short-term outcomes, and this possibility needs to be considered when interpreting our findings.

In summary, our study resulted in several interesting observations despite a modest cohort size. Applying also the Gupta definition resulted in identification of additional infants with AKI. Although a significantly larger proportion of infants required inotropic support in both the nKDIGO-AKI and Gupta-AKI groups when compared to AKI-negative infants defined by the respective criteria, other short-term outcomes did not differ significantly between groups. While evidence of long-term kidney sequelae was limited in our cohort, a history of Gupta-AKI was associated with a significantly higher median cystatin C level at 10–12 years compared to children without such a history. The clinical relevance of this finding has yet to be elucidated. To the best of our knowledge, this is the first study to report long-term outcomes following neonatal AKI defined by the Gupta criteria. Given that the existing evidence in this area is primarily drawn from small observational studies, our data contribute valuable insights to this evolving field.

In conclusion, incorporating expected postnatal SCr decline alongside the established nKDIGO definition may contribute to improved recognition of early onset neonatal AKI in near-term and term infants. The optimal approach to identifying neonates at risk of adverse neonatal outcomes and long-term sequelae, including impaired kidney function and neurodevelopment, remains uncertain. Future research should focus on refining AKI definitions to improve risk stratification and developing evidence-based protocols for monitoring and follow-up.

## Supplementary Information

Below is the link to the electronic supplementary material.

Graphical abstract (PPTX 155 KB)

## Data Availability

Data are available on reasonable request. Deidentified participant data, data dictionary, study protocol, participant and parental letter of information and written consent form are available to investigators whose proposed use of the data has been approved by an independent ethics review committee for this purpose.
